# The ‘Double Fuzzy Set’ Approach to Multidimensional Poverty Measurement: With a Focus on the Health Dimension

**DOI:** 10.1007/s11205-023-03065-1

**Published:** 2023-01-23

**Authors:** Nita Handastya, Gianni Betti

**Affiliations:** Department of Economics and Statistics, University of Siena, Siena, Italy

**Keywords:** Multidimensional deprivation, Fuzzy sets approach, Poverty

## Abstract

In more recent times, there is an increasing consensus in the field of development study to view poverty as a multidimensional deprivation beyond the more commonly used monetary perspective. Although the multidimensional poverty measurement is gaining more acceptance among policy makers, it is still based on the clear distinction between the poor and non-poor through an arbitrary threshold. One alternative to this shortcoming is offered by a multidimensional poverty measurement with a fuzzy-set approach in which it is possible to recognize deprivations as a matter of degree. The integrated fuzzy approach allows this possibility, although there is an unexplored opportunity of recognising that two or more dimensions can be attributed to a single item or attribute. This paper aims to contribute to this strand of research by introducing a ‘Double-Fuzzy’ approach. The methodology is applied to Tunisia using the Tunisian National Survey on Household Budget and Consumption (HBS) 2015.

## Introduction

Poverty is one of the biggest problems we face as a society. It is so fundamental that it is listed as one of the UN’s SDG goals. However, the discussion of the definition of poverty itself has yet to reach a consensus. One of the earliest definitions viewed poverty as a threshold representing the level of minimum bare subsistence (Rowntree, 1901). The introduction of a poverty line concept is necessary in order to move forward with the measurement of poverty.

Although it might seem like a simplification, it sets a foundation from which the study can move forward. As more attention was given to this problem, the definition expanded to also include the characteristic of dignity. Subsisting is not living; instead, an individual should achieve an acceptable living standard (Townsend, 1979). From this point, the field has grown to accept that poverty should not be characterised merely by an individual’s ability to achieve a certain income threshold.

Over the past decades, several authors have developed this issue theoretically, taking into consideration the multidimensional nature of poverty, which can be only partially captured by a single indicator, which is usually based on income or consumption (see Atkinson & Bourguignon, 1982; Bourguignon & Chakravarty, 1999; Atkinson, 2003, among others).

More specifically, poverty is a complex problem that often manifests itself in a multidimensional framework. This notion is instrumental since individuals may suffer from a different deprivation profile across a geographical context. Notably, developing countries might face several fundamental problems, such as access to water, healthcare, and education, which are more widely available in the developed world (Anand & Sen, 1997).

Various empirical works have shown that income poverty might not give us the complete picture. Chilean data shows that income does not convey a comprehensive view of poverty very well (Ruggeri-Laderchi, 1997). Indian data, instead, indicates that 53% of malnourished children do not live-in income-poor households (Steward et al., 2007).

As the field of multidimensional poverty increasingly becomes the norm, it is essential to measure the various dimensions and factors involved. Thus, it is necessary to ensure that the individual has achieved the minimally accepted levels of these attributes (Sen, 1992). Measurement mechanisms such as the multidimensional poverty index (Alkire & Foster, 2011) have become widely accepted for approaching various policy issues. However, the dichotomy between poor and non-poor remains at the centre of the discussion. Although it offers a more straightforward explanation of the current situation, it is evident that such a clear-cut division causes a lack of information and eliminates the nuances that exist between the two extremes of substantial welfare on one end and material hardship on the other (Belhadj & Limam, 2012). To deal with this issue, it is necessary to explore an alternative approach.

Based on the fuzzy set theory (Zadeh, 1965), Cerioli and Zani (1990) introduced the fuzzy set approach in poverty analysis to overcome many of the problems listed above. Since then, many authors have made seminal contributions, including on multidimensional poverty. In particular, Clark and Qizilbash (2002) innovated by introducing the distinction between vertical and horizontal vagueness; continuing with Deutsch and Silber (2005) and the book edited by Lemmi and Betti (2006) by describing the philosophical, mathematical and axiomatic aspects of the fuzzy methods; until the last contributions of Fattore (2016) and Fattore and Arcagni (2019), based on Fattore and Maggino (2014).

All these methods define at least three main steps: (i) the definition of the poverty indicator for each item of poverty or deprivation; (ii) the identification of the dimensions; (iii) the aggregation of items within each dimension. In some of these methods, including the present contribution, there is an additional step: (iv) aggregation over dimensions. This step is fully described in Clark and Qizilbash (2002) with the characteristics of horizontal vagueness. On the other hand, some other contributions, such as Fattore (2016) and the family of the posetic approach, are non-aggregative, which is their peculiarity.

In the present paper, we identify an important gap in such literature: assigning an indicator to one single dimension is often difficult. For instance, consider items/variables such as a leaking roof or mould in the walls. Do they belong to the dimension of housing conditions or environmental problems? Probably they belong to both and have different causes that vary to different degrees, representing membership functions in terms of fuzzy sets. Another example is the construction of fuzzy multidimensional Sustainable Development Goals (SDGs). Even in the construction of the traditional SDGs, some “targets” may belong to more than one SDG.^1^ Moreover, within data-driven approaches, the results from factor analyses are often doubtful or not clear at all.

We aim to close this gap with an original methodological contribution within the broader approach of fuzzy set methods: we have named it ‘the Double Fuzzy Set’ methodology (DFS), which is fully presented in Sect. 3. Before that, in Sect. 2, we outline the characteristics of the broader fuzzy set approach and the evolution of its incorporation into the literature on multidimensional poverty measurement. These brief explanations serve as an introduction to the proposal of our methodology, in which we permit that one single poverty item/indicator belongs to more than one dimension with different degrees of membership.

The paper also has a second important contribution, but, in this case, on the empirical side. This contribution is reported in Sect. 4 and consists of applying the DFS to the Tunisian Household Budget Survey 2015. This dataset is interesting for at least three main reasons. First, it allows empirical analysis linking several dimensions of quality of life that reflect a developing country. Second, an established part of the literature on the fuzzy approach to poverty measurement has been done on this dataset, thanks to works by Besma Belhadj and co-authors. Third, the increasing interest from the World Bank in developing poverty measures in Tunisia at a regional level has led to a survey conducted in 2021 on a larger sample size base (Betti et al., 2021).

Finally, the last section of the paper concludes and proposes further developments of the methodology and its application in the social sciences.

## The Fuzzy Sets Approach and Poverty Measurement

The fuzzy set approach was firstly introduced in the field of computer science. It is based on the idea that there are conditions in which the full truth cannot be observed. The world is complex, and sometimes it is not possible to assign an observation to a particular set with full certainty. To understand this better, the most straightforward analogy would be age. A person cannot be assigned entirely to a category of old or young, instead, they are on a degree of oldness or youngness. Fuzzy logic is suitable for handling such partial truth.

Moreover, fuzzy sets “… welcome a certain degree of ambiguity that is present in several social science constructs….” (Henriques et al., 2018), and thus they permit to properly incorporate measurement errors intrinsically connected with the “ambiguity” of subjective multidimensional assessment of poverty.

Instead of approaching matters with the traditional crisp and Boolean logic that requires an absolute membership to only one set (i.e., 1 OR 0), the fuzzy set approach acknowledges that there is a gradual transition between the extreme (i.e., between the interval [0, 1]). This idea is compatible with the reality of poverty, which is not simply present (= 1) or absent (= 0); instead, it exists as a degree. The theory itself was proposed by Zadeh (1965), but the attempt to incorporate it into the multidimensional poverty framework was made much later by Cerioli and Zani (1990).

Once the identification of items to be included in the indices is performed in the first, they define a quantitative membership function in the range [0,1], determined for each item j, as follows:

1 $${\mu }_{j}({x}_{ij})=\left\{\begin{array}{ll}0& {{x}_{ij}=min(x}_{j})\\ \frac{{\mathrm{max}({x}_{j})-x}_{ij}}{\mathrm{max}({x}_{j})-\mathrm{min}({x}_{j})}& {min(x}_{j})<{x}_{ij}<{max(x}_{j})\\ 1& {{x}_{ij}=max(x}_{j})\end{array}\right.$$

where min(x_j_) is the category associated with the lower poverty level, and max(x_j_) is the category associated with the highest poverty level.

Since then, there have been two main periods, defined as *development* and *expansion*. In fact, the method was further developed by Cheli and Lemmi (1995), which resulted in the Totally Fuzzy and Relative (TFR) approach. The development of the study did not stop there, as it was later refined by Betti et al. (2006), which yielded the Integrated Fuzzy and Relative (IFR) approach. The book by Lemmi and Betti (2006) concluded this first period of development with the definition of an overall framework based on the philosophy of fuzzy sets, on mathematics through an axiomatic approach, and on various economic aspects.

From 2006, as the new period of *expansion*, the fuzzy method has moved in several directions. First, through the introduction of the fuzzy approach, two additional factors are to be decided: (i) the choice of the membership function and (ii) the choice of rules for manipulating the resulting fuzzy sets. The former refers to the quantitative specification of the deprivation risk faced by individuals based on their respective populations and the diverse non-monetary aspects that determine their living conditions. The latter focuses on a more technical counting aspect concerning intersections, unions, and averaging of the sets (Betti & Verma, 2008).

Betti et al. (2006) used a more generalised form of the membership function which defined deprivation suffered by individual $i$ as:

2 $${\upmu }_{i,K}={\left(\frac{{\Sigma }_{\gamma =i+1}^{n}{w}_{\gamma }|{X}_{\gamma }>{X}_{i}}{{\Sigma }_{\gamma =2}^{n}{w}_{\gamma }|{X}_{\gamma }>{X}_{1}}\right)}^{{\alpha }_{K}-1}\left(\frac{{\Sigma }_{\gamma =i+1}^{n}{w}_{\gamma }{X}_{\gamma }|{X}_{\gamma }>{X}_{i}}{{\Sigma }_{\gamma =2}^{n}{w}_{\gamma }{X}_{\gamma }|{X}_{\gamma }>{X}_{1}}\right)$$

where $X$ is the equivalised income in the monetary deprivation, ${w}_{\gamma }$ is the sample weight of individuals of rank $\gamma$ in the ascending income distribution, and ${\alpha }_{K}$ $(K=1, 2)$ are two parameters corresponding respectively to monetary and non-monetary deprivation. The monetary-based indicators are then defined as Fuzzy Monetary (FM) while the non-monetary based ones are defined as Fuzzy Supplementary (FS).

The duality allows for a composite measure encompassing non-monetary deprivations such as education, health, and living standard (housing, etc.) without completely disregarding the importance of monetary deprivation.

Once the identification of dimensions in the second step is performed, the aggregation of items or their membership functions within each dimension constitutes a crucial step in constructing multidimensional poverty indicators. The literature is rich in methods for the calculation of such aggregating weights. In general, these weighting methods capture two aspects: the “prevalence weights”, which accounts for the statistical dispersion of the items; and the “correlation weights”, which accounts for the correlation with other items in the same dimension.

Betti and Verma (2008) proposed one weighting system which considers both aspects: the dispersion of a poverty item and its correlation with other items in the given dimension. This method can be represented as $w_{hj} = w_{hj}^{a} \cdot w_{hj}^{b} ,h = 1,\,2,\,...,\,m;j = 1,\,2,\,...,\,k_{h}$, where *h* is a generic dimension and *j* a generic poverty item. The first element of this equation is the coefficient of variation of the complement of 1 of the membership function’s value of an item, specified as follows:

3 $$w_{hj}^{a} \propto \frac{{std_{hj} }}{{1 - mean_{hj} }}$$

The second element, defined as a measure of the correlations, can be computed in the following form:

4 $$w_{hj}^{b} \propto \left( {\frac{1}{{1 + \sum\limits_{j = 1}^{{k_{h} }} {r_{{e_{{hj,hj^{^{\prime}} }} }} |r_{{e_{{hj,hj^{^{\prime}} }} }} < r_{{e_{hj} }}^{*} } }}} \right) * \left( {\frac{1}{{1 + \sum\limits_{j = 1}^{{k_{h} }} {r_{{e_{{hj,hj^{^{\prime}} }} }} |r_{{e_{{hj,hj^{^{\prime}} }} }} < r_{{e_{hj} }}^{*} } }}} \right)$$

where $r_{{e_{{hj,hj^{^{\prime}} }} }}$ is the correlation coefficient between items j and j’ in the h-dimension and $r_{{e_{hj} }}^{*}$ is a critical value of the correlation coefficient (a detailed and technical definition of such critical value can be found in Betti & Verma, 2008).

Empirical results on multidimensional poverty have been produced based on the fuzzy approach, notably in the countries of the European Union (Betti et al., 2015), which have identified a well-established set of dimensions. From that point, the fuzzy set method has been extended to other fields in the social sciences, namely to the quality of life, violence against women, employment status, and educational mismatch, which have been well summarised by Betti and Lemmi (2021).

## The New ‘Double Fuzzy Set’ Approach (DFS)

More recently, with data availability, it is possible to develop the fuzzy methodology further. For instance, questions asked in a survey are often attributable to more than one dimension. This fact highlights the multidimensional nature of poverty and how attributes are commonly interlinked. By assigning a response to only one dimension and ignoring the possibility of a second or even third dimension, we risk losing a layer of observation. Based on this notion, we attempt to further refine the current state of the art in fuzzy poverty measurement by introducing the double fuzzy set methodology. In this proposed method, we explore a second area where the concept of degree can be identified instead of a simple presence or absence that takes place in the Fuzzy Supplementary measures.

**Fig. 1:**
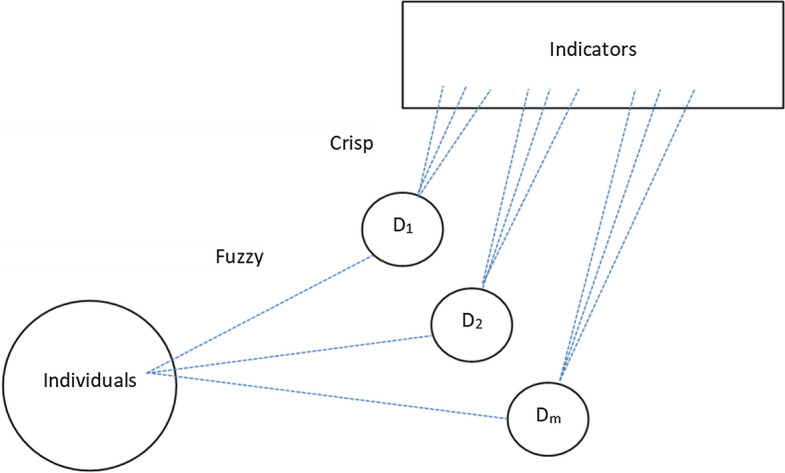
Step (ii) in multidimensional fuzzy approaches

To explain the method in detail, it is useful to recall that the most innovative contributions in multidimensional poverty measurement (both fuzzy and crisp) adopt step-by-step procedures. These studies usually include at least three main steps: (i) the definition of the poverty indicator for each item *j: 1, …, J* of poverty or deprivation over the *i*: 1, …, *n* individuals or households; (ii) the identification of the *M* dimensions; and (iii) the aggregation of items within each dimension. Our original proposal makes innovations within step (ii). Different from our method, previous methodological proposals assign each indicator to one and only one dimension (see Fig. 1), even when some indicators are highly correlated with other dimensions. We define these methods as “crisp”, recalling the traditional poverty analysis in which each individual is assigned to either the crisp set of poor or non-poor people.

As elaborated in the previous section, the crisp assignment of an indicator to only one dimension can be misleading or inaccurate because it disregards the fact that it might be related to another dimension. For instance, an item of ‘leaking roof’ or ‘mould in walls’ can belong to both ‘housing conditions’ and ‘environmental problems’ dimensions. However, by assigning them to only one dimension, we risk losing important information, especially in the context of poverty measurement. Another emerging example in the recent literature is the construction of fuzzy multidimensional Sustainable Development Goals (SDGs), where the “targets” may belong to more than one SDG. In this case, the DFS approach can also be very useful.

**Fig. 2:**
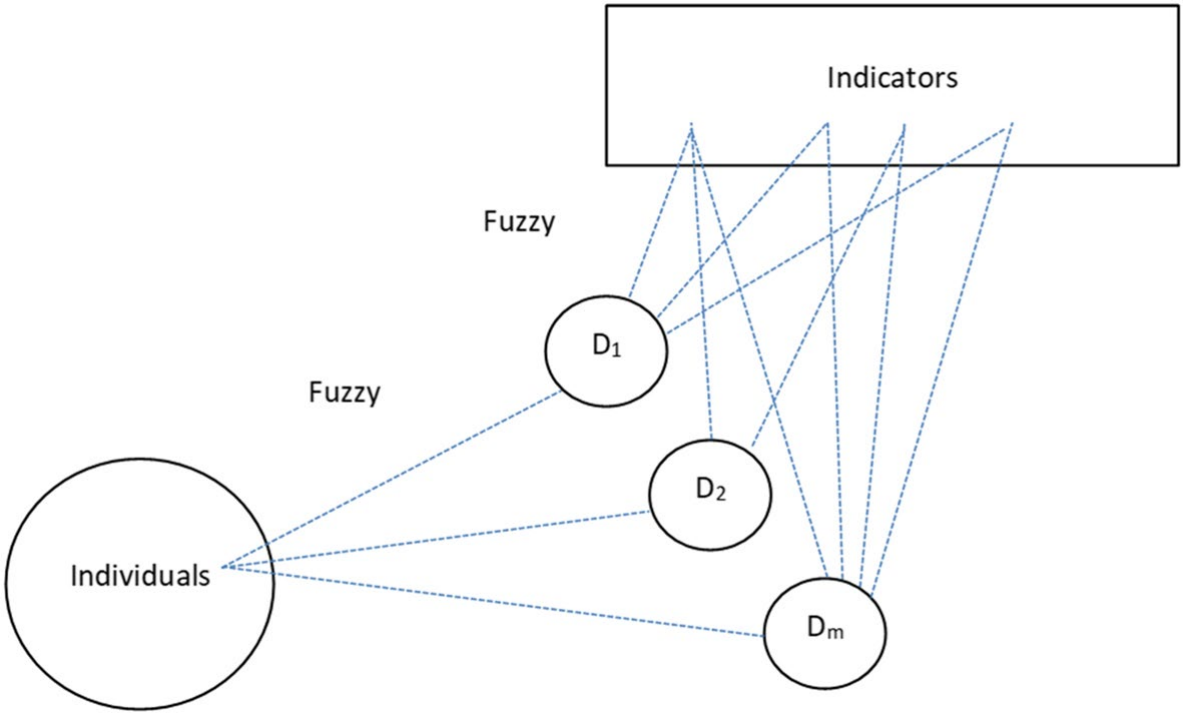
The double fuzzy set approach (DFS)

In order to solve these problems, we propose an approach where we introduce the second aspect of fuzziness in the membership function for each indicator: we assign these indicators to more than one dimension resulting in various membership degrees. This assignment is illustrated well in Fig. 2, where several lines from each indicator go to one or more dimensions. From a technical and mathematical point of view, we propose a set of *M* membership functions for each indicator j (j: 1, 2, …, J), one for each dimension described in Fig. 2. These dimensions could be seen as a set of M fuzzy states, and the corresponding membership could be written as follows^2^:

5 $${\upmu }_{j,m}\in {\left(\mathrm{0,1}\right)} with \sum_{m:1}^{M}{\upmu }_{j,m}=1$$

Given the proposed method, we can now define the proposal of Cerioli and Zani (1990) in Eq. (1) as Single Fuzzy Set (SFS) methodology. In the same way as in Cerioli and Zani (1990), we can use different membership functions while using the DFS method. The membership function in Eq. (2) is a possibility, but authors may choose the membership function that best fits their purposes.

In this paper, we propose to adopt the results obtained in the identification of the M dimensions in step (ii) performed with multivariate statistical methods, in particular the exploratory factor analysis (EFA) firstly proposed by Whelan et al., 2001, and later implemented in Eurostat (2002) and Betti et al. (2006). In such SFS approaches, these factor analyses are performed in order to assign each indicator (often referred to as individual variables or items) to one specific dimension within the multidimensional approach.

In our proposal, the membership function ${\upmu }_{j,m}$ of the DFS in Eq. (5) is determined based on the factor loadings ${\uplambda }_{j,m}$ of each indicator *j* in any factor (dimension) *m*. From these loadings, the membership functions ${\upmu }_{j,m}$ are defined as:

6 $${\upmu }_{j,m}=\frac{{\uplambda }_{j,m}}{\sum_{m:1}^{M}{\uplambda }_{j,m}}$$

For each indicator j, any membership function ${\upmu }_{j,m}$ represents the degree of each dotted line reported in Fig. 2 linking the indicator j and the dimension D_m_ (m: 1, 2, … M).

An alternative proposal could be based on the “correlation” in line with the principle contained in Eq. (4). Such correlations could be used in two steps: in the first step, the correlation between an item j and a dimension m ${\uprho }_{j,m}$ is used for calculating the membership function ${\upmu }_{j,m}$ as follows:

7 $${\upmu }_{j,m}=\frac{{\uprho }_{j,m}}{\sum_{m:1}^{M}{\uprho }_{j,m}}$$

In the second step, the correlations are used in order to calculate the *relative* weight of each item j in a specific dimension m, as the product of ${\mathrm{w}}_{j,m}$ * ${\upmu }_{j,m}$.

## Empirical Illustration of the DFS Approach

In this section, we present the new Double Fuzzy methodology based on the 2015 Household Budget Survey conducted in Tunisia. As already highlighted in the introduction, this is an interesting data base to be used as a case study for at least three main reasons:

(i) There is a vast body of literature on the fuzzy set approach to poverty measurement that uses this dataset. This is due to the research of Prof. Besma Belhadj and co-authors, based on the 2015 HBS in Tunisia or previous surveys.
(ii) There is increasing interest from the World Bank in exploring poverty measures in Tunisia at the regional level, which has led to a new survey conducted in 2021with a larger sample size (Betti et al., 2021). Such interest is also present in Algeria, with the new Household Budget Survey conducted in early 2022.
(iii) There is an emerging literature that aims to analyse the effect of COVID-19 on several dimensions of quality of life. Tunisia could be the first African country to apply Tavares and Betti’s (2021) new method.

As already mentioned, studies on multidimensional and fuzzy poverty in Tunisia have multiplied in the last decade. Belhadj (2011) firstly analysed multidimensional poverty using fuzzy set theory, applied to Tunisian data from the 1990 budget and consumption survey. Later, Nasri and Belhadj (2017) used household expenditure, considering consumption for only three pillars: food, health, and education. For other seminal contributions, see, among others, Belhadj (2012), Zedini and Belhadj (2015), and Nasri and Belhadj (2018).

This section begins with a brief description of the so-called traditional approach to poverty measurement in Tunisia, which constitutes the benchmark for the second sub-section on fuzzy measures.

### The Traditional Poverty Approach in Tunisia

The choice of a (traditional) poverty measure determines how a set of factual information is perceived. Although it is tempting to draw a line that divides the population, there is the risk of overlooking the reality of those who are located around the threshold, moving between the two groups based on an arbitrary cut-off. To illustrate this point, observe Table 1 below on the poverty level in Tunisia over time, based on different thresholds.

**Table 1:** Comparison of poverty thresholds in Tunisia

	2000 (%)	2005 (%)	2010 (%)	2015 (%)
National	25.4	23.1	20.5	15.2
International ($1.90 PPP)	6.0	3.4	2.0	0.2
Lower middle-income class ($3.20 PPP)	22.9	15.2	9.2	3.0
Upper middle-income class ($5.50 PPP)	50.6	41.5	30.5	17.5

Although the four measures show the same trend with a declining poverty rate over the past two decades, their scale is of different magnitudes. Take the international poverty line, with a threshold of $1.90. We started at 6% in 2000 and arrived at 0.2%, a 97% decrease.

While it sounds encouraging, the starting point might lead us to believe that there is very little incidence of poverty in Tunisia in the first place. Furthermore, the endpoint gives the impression that poverty might have been eradicated. It is dangerous to assume this, especially if the threshold is meant as a building block of the policy-making process that otherwise would be able to improve the lives of many people.

Meanwhile, at the same time, the national poverty line^3^ indicates a different story. It starts with roughly a quarter of the population below the poverty line and reached 15.2% in 2015, a decrease of 40%. This figure is less than half of the percentage of decrease that the international poverty line shows. It is much more representative of the reality due to the selection carried out by the officials, which differentiates between spatial units. The cost of living varies across geographical locations, and this should be taken into consideration when drawing such a line. Given the same amount of money, those who live in a rural area would be able to stretch it out for a longer period compared to their peers in a metropolitan area due to price differences. It is possible to increase the line if needed, as shown by the two additional measures.

The thresholds are supplied by the World Bank dataset, pegged at the level of $3.20 PPP and $5.50 PPP, respectively. Both showed a rather sharp decline in poverty, with the latter putting half of the population under the poverty line, almost twice that reported by the national threshold. Thus, the measurement choices present some dilemmas to the data users: one might overestimate, while another may underestimate. Although the national and middle-upper class thresholds present a more believable story, it does not address the instability risk of those who are located around the cut-off.

In addition, the poverty line is largely perceived from a monetary point of view. This is no longer adequate, especially since the multidimensional poverty concept has gained wider acknowledgement. The financial situation provides one side of the story, but it is not the whole story.

### The Double Fuzzy Set approach in Tunisia

This subsection describes the implementation of the fuzzy set approach in Tunisia. This firstly consists of calculating the fuzzy counterpart of the headcount ratio, namely the Fuzzy Monetary measure introduced in Sect. 2; then, the calculation of the Double Fuzzy Set approach with six Fuzzy Supplementary measures.

**Fig. 3:**
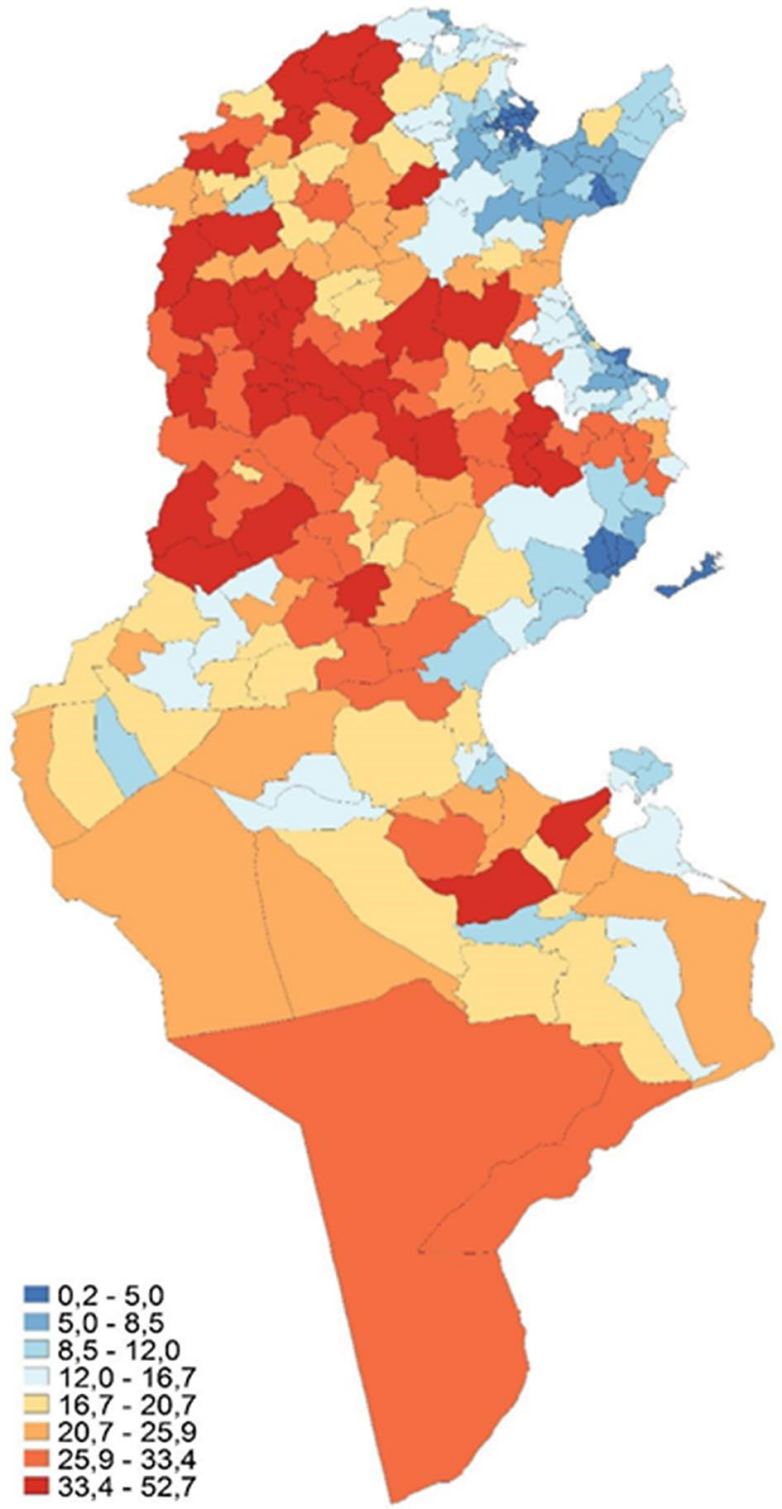
Poverty rate across Tunisia 2015

**Fig. 4:**
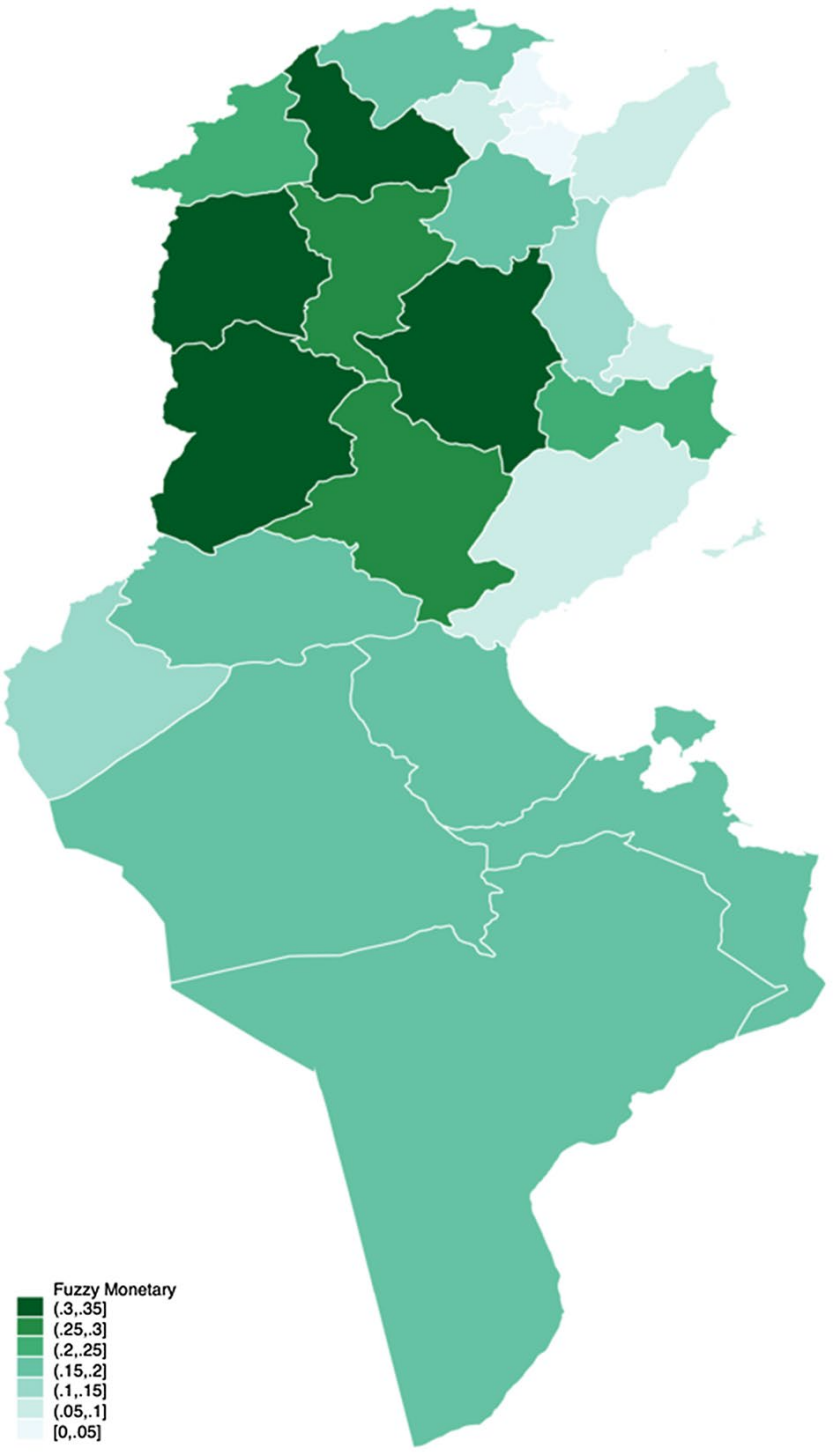
Fuzzy monetary

**Fig. 5:**
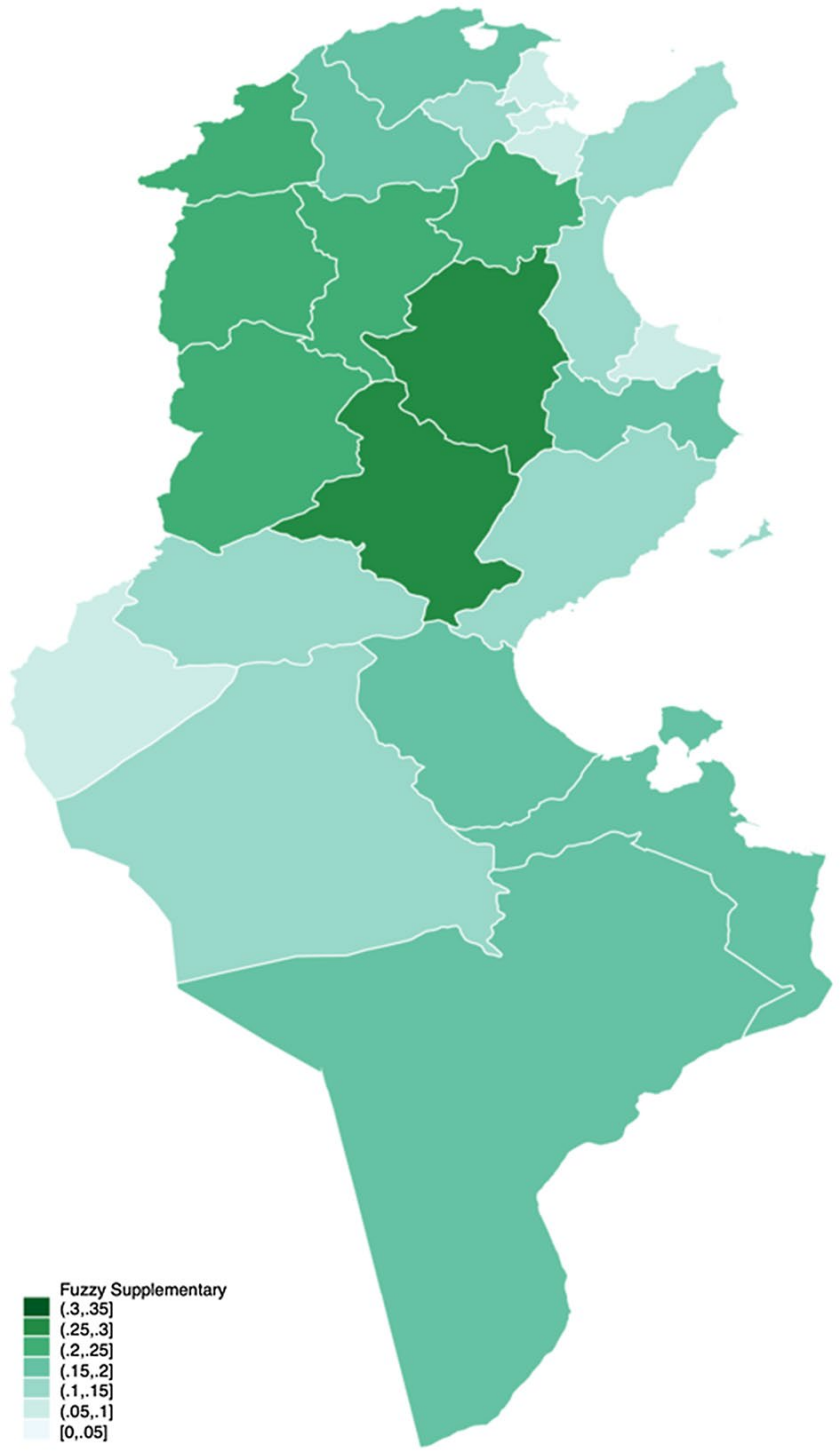
Fuzzy supplementary

Figure 4, on the left, indicates the fuzzy monetary approach, while on the right, Fig. 5 illustrates the fuzzy supplementary approach. Notice that the different shades represent the poverty degrees as presented with fuzzy measures, not the poverty incidence percentage as presented in Fig. 3.

The concentration of a darker shade in the northwest coincides with both fuzzy monetary and supplementary. We can see a slight variation between the two, especially regarding the intensity. From Fig. 4, the fuzzy monetary perspective, the governorates that are doing worse are Le Kef, Kasserine, Béja, and Kairouan. However, it seems that, in general, there is a clear difference between the northwest and the centre-west with the rest of the country. This is consistent with the findings of the National Institute of Statistics (2012), in which both regions exhibited the highest poverty rate in the country, followed by the southeast and the southwest regions. The same theme can be seen in Fig. 5, the fuzzy supplementary perspective, although the magnitude is not as pronounced as in the monetary approach.

Fuzzy supplementary is based on the multidimensional approach to poverty, and thus can be disaggregated. In building the indicator, we found six major dimensions to be studied: health, education, work, housing, durables, and distance. They form a picture of the necessities for a dignified life. However, since we allow items of the survey to be assigned to a secondary set, an additional layer of observation can be obtained. The difference between this approach with the previous one is in this step of assignation to the second dimension. Whereas the SFS methods assign each item to one dimension, we consider a second suitable dimension.

**Fig. 6:**
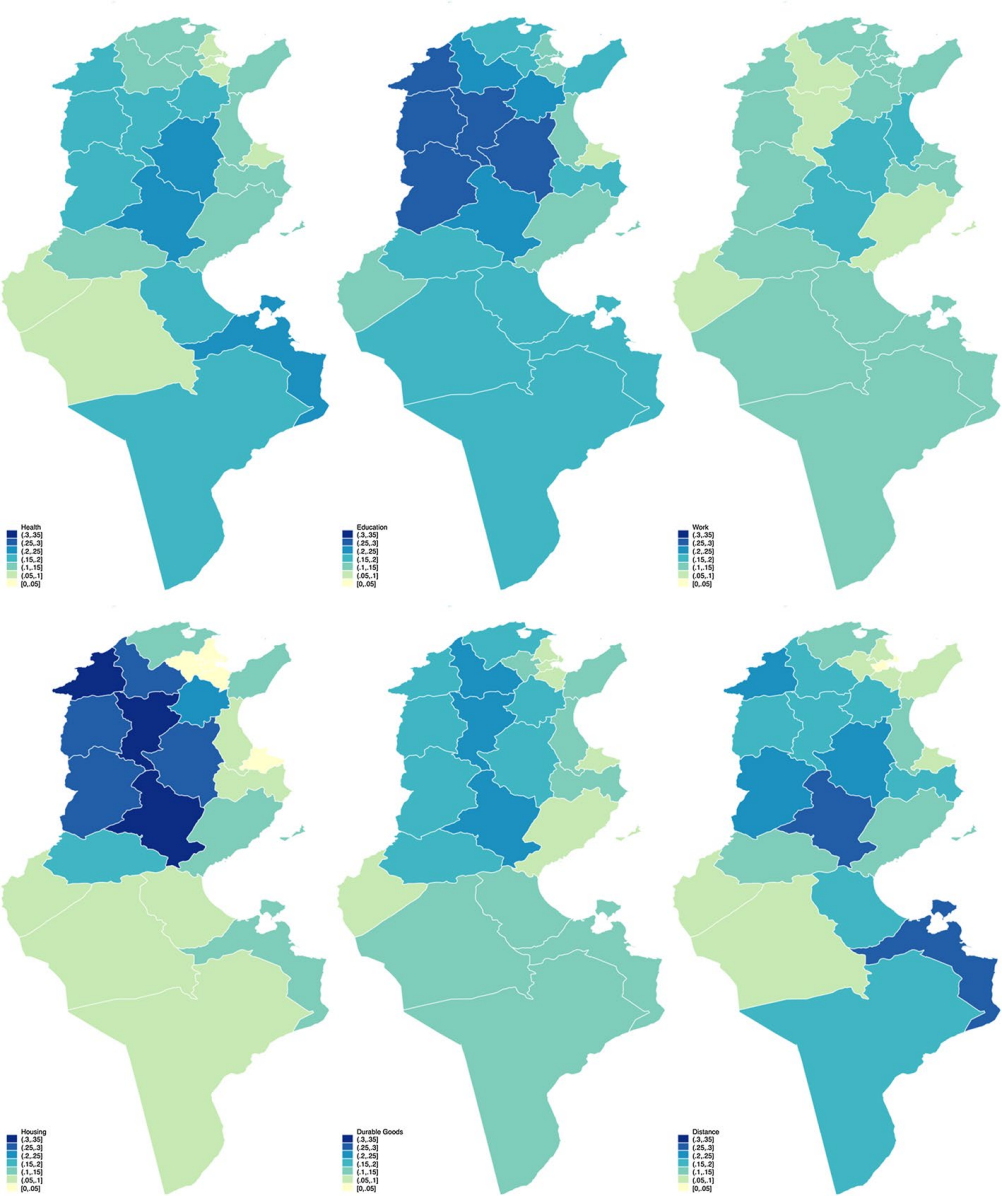
Fuzzy supplementary: the six dimensions

For example, survey questions regarding access to health, work, and education asked why an individual was deprived of these services. Although the reasons may vary, the common theme of distance emerged. This fact highlights the divide between urban and rural areas and allows us to take a glimpse into the spread of deprivation across the country. The maps in Fig. 6 portray the six categories we discovered in the Tunisian HBS.

The first category is health. Most Tunisian have a form of access to healthcare. Tunisia boasts a low infant mortality rate compared to the rest of Africa. However, there is a divide between regions regarding this matter. Infant mortality is higher in the central west and south compared to the rest of the country (Africa Development Bank, 2014). There are several possible explanations for this. Although social security coverage is available, the availability of medical practitioners is at various levels among governorates, favouring those on the coasts: Tunis, Sousse and Monastir (on the central east, also known as the Sahel). Income inequalities also play a part in this narrative. As out-of-pocket health expenditure is still required, the monetary disadvantage means that the incidence of deprivation can be a problem in low-income regions. The inequality across the governorates is not unique to health, as we will explain further.

The second category is education. Tunisia has quite a high literacy rate, with mandatory primary education in place. However, we can also see inequalities among regions, with the north and central west suffering the most. There is an issue with access to higher education due to its high demand. Universities are clustered in the coastal areas, especially in Tunis, whereas there are barely any universities in the other parts of the country.

This incidence permeates to the third category, work availability. Internal migrations from the inner part of the country to the coastal area, especially to Greater Tunis (Tunis, Ariana, Ben Arous, and Manouba), is a major phenomenon (Amara & Jemmali, 2018).

This problem can be attributed to uneven investments among regions. While the north and central west are abundant with natural endowments, development has largely been focused on those around the Sahel or the central east, creating a division between the least favoured and the most favoured region in almost all aspects (Africa Development Bank, 2014).

In addition, the natural endowments do not seem to improve local life since it is used to improve the Capital. As the dimensions are interlinked, it is not surprising to find that the same pattern emerges in the fourth and fifth categories: housing and durable goods.

It seems that the unequal distribution of investment creates a wide regional divide, not only between rural versus urban, but, more specifically, between the Sahel region versus the rest of the country (Table 2).

**Table 2:** Poverty measures at Governorate level

Governorate	Size	HCR	FM	FS	FS1	FS2	FS3	FS4	FS5	FS6
					Health	Education	Work	Housing	Durable	Distance
ARIANA	4144	0.054	0.046	0.093	0.088	0.121	0.109	0.046	0.085	0.064
BEJA	2591	0.320	0.308	0.193	0.125	0.231	0.089	0.278	0.201	0.177
BEN AROUS	4316	0.043	0.046	0.084	0.065	0.105	0.119	0.016	0.061	0.069
BIZERTE	4321	0.175	0.189	0.164	0.104	0.173	0.127	0.136	0.165	0.110
GABES	5514	0.158	0.164	0.150	0.155	0.178	0.122	0.066	0.124	0.194
GAFSA	5702	0.180	0.181	0.149	0.117	0.156	0.120	0.195	0.157	0.119
JENDOUBA	4431	0.224	0.237	0.237	0.190	0.265	0.118	0.310	0.164	0.217
KAIROUAN	5665	0.349	0.341	0.259	0.217	0.264	0.182	0.252	0.176	0.216
KASSERINE	5425	0.328	0.311	0.244	0.196	0.273	0.133	0.252	0.181	0.237
KEBILI	6197	0.186	0.196	0.115	0.071	0.165	0.119	0.085	0.109	0.061
LE KEF	3740	0.342	0.313	0.215	0.152	0.258	0.113	0.283	0.167	0.181
MAHDIA	2574	0.211	0.236	0.175	0.114	0.186	0.145	0.066	0.147	0.166
MANOUBA	2241	0.121	0.097	0.135	0.117	0.153	0.150	0.031	0.101	0.093
MEDENINE	5447	0.216	0.199	0.171	0.230	0.167	0.140	0.103	0.141	0.265
MONASTIR	2393	0.083	0.059	0.079	0.098	0.094	0.124	0.026	0.066	0.074
NABEUL	4085	0.074	0.094	0.145	0.119	0.166	0.131	0.135	0.131	0.085
SFAX	5051	0.058	0.080	0.115	0.113	0.147	0.092	0.114	0.085	0.119
SIDI BOUZID	5696	0.231	0.253	0.265	0.250	0.247	0.172	0.328	0.233	0.266
SILIANA	2403	0.277	0.273	0.219	0.155	0.272	0.087	0.309	0.207	0.165
SOUSSE	4660	0.161	0.136	0.134	0.107	0.132	0.151	0.053	0.147	0.100
TATAOUINE	6590	0.149	0.159	0.161	0.166	0.192	0.137	0.080	0.126	0.189
TOZEUR	5543	0.146	0.148	0.089	0.092	0.122	0.089	0.074	0.083	0.085
TUNIS	3798	0.035	0.034	0.098	0.082	0.124	0.131	0.015	0.074	0.041
ZAGHOUAN	2483	0.122	0.167	0.206	0.160	0.232	0.124	0.246	0.162	0.179

There seems to be a strong clustering of infrastructure investment around the Greater Tunis area. This brings us to the final dimension, which we observe to be interlinked with almost all five previous categories: distance to services. The divide also means that jobs, educations, and health services are more accessible around the capital than in the rest of the country, which amplifies other dimensions in the measurement. When combined, the six dimensions provide a picture of multidimensional deprivations that complement the poverty line approach by adding a layer of observation.

For the visualisation, we have decided to use a uniform bracket. This results in an across-dimensions observation in which we can compare the degree of deprivation between them. It is very noticeable that housing is the dimension that exhibits the most inequality, with severe deprivation found in the north and central-west regions. The housing situation is central to many aspects as it determines the family's living conditions.

Poor housing can be a source of ill health and the cause of a host of problems that would not allow individuals to achieve their full potential. It is followed by education, which we suspect to manifest because there are very few higher education institutions outside the Greater Tunis area. An indication that there is insufficient investment in this dimension would mean that those who can afford it would migrate to study elsewhere or simply drop out of school.

One study highlighting the theme of internal migration in Tunisia indicates this issue, which might be why the dimension of work does not seem to show a contrast between regions (Amara & Jemmali, 2018). It is because those who could afford to move have migrated. Education is then followed by distance, which indicates a mobility challenge to access basic services, durable goods and health, which show a similar pattern and intensity.

## Concluding Remarks

During the last decade, the Fuzzy Set approach has been developed and extended to other social fields: among others, marital disruption (Aassve et al., 2007), violence against women (Bettio et al., 2020), financial literacy measurement (Hizgilov & Silber, 2021), and labour employment (Belhadj & Kaabi, 2021). The book by Betti and Lemmi (2021) provides an exhaustive list of such topics. In this context, the double fuzzy approach could have further applications: an example could be the inclusion of individual items into more than one dimension, as in the case of SDG targets in more than one SDG.

Moreover, since the single fuzzy approach measures provide smaller standard errors than traditional or “counting” approaches, the introduction of the double fuzzy may further reduce these standard errors, which will be a future area of research, following the line of research carried out by Betti et al. (2018). In conclusion, the DFS approach may improve the measurement of poverty in at least three aspects, as highlighted in Table 3:

(i) Lack of arbitrariness: compared with the traditional approaches, there is no need to define an arbitrary poverty line. Moreover, compared to the IFR approach, there is no need to (it is sometimes arbitrary) assign any indicator to one and only one specific dimension.
(ii) (Statistical) precision: standard errors would be greatly reduced compared with the traditional approach, in line with Betti et al. (2018).
(iii) Dimension completeness: each dimension of the DFS approach can incorporate as many individual items as possible so as to maximise its complete description of the phenomenon under investigation.

**Table 3:** Properties of various poverty approaches

	Traditional	IFR	DFS
Lack of arbitrariness	NO	VERY GOOD	EXCELLENT
(Statistical) precision	GOOD	VERY GOOD	EXCELLENT
Dimension completeness	NO	VERY GOOD	EXCELLENT

## Footnotes

1 https://sdgs.un.org/goals.

2 Indicators refer to the survey items such as ‘type of walls’, while dimensions refer to a group of survey items such as ‘housing condition’.

3 The national poverty line is determined by the National Institute of Statistics (INS) to define the minimum standard of living in the country. The welfare aggregate is spatially adjusted to mirror the requirements in different spatial units. There are three poverty lines in Dinars (TND) per person per year: Metropolitan areas (TND 1878), Communal (TND 1703), and Non-Communal (TND 1501). On average, the poverty line is at the level of TND 1706, or roughly $2.50 USD/PPP.
